# Factors Associated with Unprotected Anal Sex among Men Who Have Sex with Men in Mexico

**DOI:** 10.3390/idr14040058

**Published:** 2022-07-21

**Authors:** Ramiro Caballero-Hoyos, Joel Monárrez-Espino, María Guadalupe Ramírez-Ortíz, Francisco Martín Cárdenas-Medina

**Affiliations:** 1 Clinical Epidemiology Research Unit, Mexican Institute of Social Security, Colima 28040, Mexico; jose.caballeroh@imss.gob.mx; 2 Department of Health Research, Christus Muguerza del Parque Hospital, University of Monterrey, Chihuahua 31000, Mexico; 3 Medicine and Health Sciences Unit, Zacatecas Autonomous University, Zacatecas 98000, Mexico; 4 Manzanillo Social Security Center, Mexican Institute of Social Security, Manzanillo 28210, Mexico; ishaoro@hotmail.com; 5 VIHda Manzanillo, Private Assistance Institution, Manzanillo 28210, Mexico; martinmanzanillo@gmail.com

**Keywords:** anal sex, HIV-AIDS, MSM, stigma, syndemic

## Abstract

The global prevalence of HIV is notably higher in men who have sex with men (MSM) compared with other male populations. Unprotected anal intercourse is the riskiest sexual behavior for HIV acquisition and/or transmission among this minority population. The purpose of the study was to identify if the syndemic of psychosocial stressors and experienced stigma are predictors of unprotected anal sex in Mexican MSM. A cross-sectional analytic study was carried out. It included adults residing in Manzanillo, Mexico, with oral/anal sex practices within the last year. Informed consent was given by 142 participants selected using snowball sampling. Collected data included sociodemographic characteristics, psychosocial stressors, experienced stigma, HIV knowledge, knowing a friend/acquaintance living with HIV/AIDS, and sexual risk behaviors. Adjusted logistic regression was used to identify predictors of unprotected anal sex within the last six months. Presence of syndemic of psychosocial stressors, drug use during sex, having friends/acquaintances with HIV/AIDS, and experiencing high stigma were positively associated; high level of HIV knowledge was negatively linked. Reducing psychosocial stressors and integrating stigma-mitigation strategies are key elements to reduce HIV transmission.

## 1. Introduction

Nearly four decades after the beginning of the HIV-AIDS pandemic, the global prevalence of HIV is notably higher in men who have sex with men (MSM) compared with other male populations. In this group, the sustained high incidence rates led to the continued expansion of the epidemic over time regardless of its geographic location [1]. In 2019, 62% of new adult infections occurred in specific populations including MSM, transgender people, sex workers, intravenous drug users, and prisoners, even when they constitute a small fraction of the general population [2]. Compared with heterosexuals and intravenous drug users, MSM have the highest lifetime risk for HIV, and continue to account for most new diagnoses [3].

In Latin America, the HIV epidemic is concentrated among vulnerable populations such as MSM and transgender people who live in urban areas with prevalence rates ranging from 1.2% to 32.6%, and even higher among young people under 25 years of age. In young MSM, new HIV infections have increased in number over the past years despite declining incidence in the general population [4,5]. Using surveillance surveys and rapid detection tests, HIV prevalence was 12–15% in Mexican MSM sex workers, and 10% in MSM urban residents in 2007 [6]. A survey of meeting places from 24 cities conducted in 2011 found an HIV prevalence of 16.9%; this is 73 times higher than that of adults of reproductive age [7]. It has also been reported that HIV vulnerability of MSM is exacerbated when these men reside in contexts where stigma, homophobia, and discrimination prevail against them as a population minority [8].

Apart from the injection of drugs, unprotected anal intercourse is the riskiest sexual behavior for HIV acquisition and/or transmission in MSM [9]. According to a British survey of sexual attitudes and lifestyles, MSM showed worse perceived physical, mental, and sexual health conditions compared with the general population [10]; MSM reported higher occurrence of unprotected anal sex with more than two partners within the last year, higher prevalence of sexual transmitted infections (STI), more anxiety and depression, and more consumption of illegal drugs.

Studies from various North and South American countries [8,11,12,13,14] and China [15,16] identified risk factors associated with unprotected anal sex in MSM that can be classified into three main categories: (1). Circumstantial factors including those related to the higher frequency of sexual relations with multiple partners in different locations, the type of sex partner (main or casual), and the serologic HIV status (concordant or discordant); (2). Mental health factors, including diagnoses of depression and anxiety, and the use of alcohol or illicit drugs during sexual encounters; and (3). Cultural factors associated with the heteronormative context that leads to social stigma with subsequent feelings of guilt, higher rates of anxiety and depression, and riskier sex practices, along with poor or absent exposure to preventive HIV educational interventions. These factors can be conceptually framed into the “sexual minority stress theory” whereby homophobic environments fight against MSM lifestyles through expressions of discrimination and victimization causing perceptions of stress in daily life contributing to the increase of unprotected anal sex practices with the consequent higher vulnerability to HIV infection [17]. Two stress factors studied in MSM linked to unprotected anal sex practices are the syndemic of psychosocial stressors, and the stigma experienced by MSM.

Syndemic theory states that adverse social conditions grounded in inequality, marginalization, and political oppression lead to the development of epidemics affecting specific population groups such as MSM, resulting in adverse biological, social, or behavioral interactions with the disease [18]. Syndemic studies of psychosocial stressors in MSM showed that the additive accumulation of stressors (i.e., dose-response) was associated with an increased risk of unprotected anal sex, and with a higher incidence of HIV infections [19]. Studies including multicentric cohorts from United States [20,21,22,23], cross-sectional surveys conducted in the world [24] and Latin-American [25] populations, and in local samples of Shanghai [26] and Philadelphia [27] also found that cumulative psychosocial stressors (e.g., anxiety, depression, illegal drug use, sexual childhood abuse, partner violence, sexual compulsivity, etc.) predicted the occurrence and/or increase the risk of unprotected anal sex and HIV infections.

In terms of experienced stigma and unprotected anal sex, the classical definition by sociologist Erving Goffman conceives stigma as the social discrediting of a marginalized individual or group by others due to perceived negative attributes [28]. In the case of MSM, stigma can relate to the sexual minority status, but also to the gender expression and HIV status. Three studies focusing on the relationship between stigma experienced by MSM and unprotected anal sex practices found various significant risk factors. A secondary analysis of HIV surveillance databases from the United States found associations between stigma and unprotected anal sex in discordant HIV partners, in MSM with many sexual partners, and in those practicing survival sex [29], and in the follow-up of a Nigerian cohort of MSM, an association was also found with casual partners after adjusting for mental health stressors and sociodemographic variables [30]. Lastly, a Chinese cross-sectional study reported that internalized stigma was associated with the risk of unprotected anal sex after adjusting for childhood physical abuse, mental health stressors, and community participation [31].

In Mexico, the study of unprotected anal sex in MSM as a risk factor for HIV infection has been mainly done using cross-sectional analyses of the adjusted effect of individual psychosocial stressors. One study found a positive association with depressive symptoms and sexual compulsivity [11], while another found it with cocaine use and with the experience of sexual abuse by MSM with recent HIV infection [32]. Just one study conducted in the border city of Tijuana approached the subject using a syndemic focus of psychosocial stressors reporting a dose-response pattern between the presence of more syndemic conditions and a higher frequency of unprotected anal sex with casual partners [33]. So far, no studies on the effect of social stigma have been published in spite of evidence from a qualitative research showing that the Mexican cultural context stigmatizes and discriminates MSM with resulting negative effects in HIV preventive and control strategies [8].

Therefore, this is the second study conducted in Mexico where a syndemic approach of psychosocial stressors is used, but the first looking at social stigma, as potential predictors of unprotected anal sex among MSM in Colima (a western coastal city that has the highest proportion of MSM in the Mexico), potentially leading to more HIV vulnerability.

## 2. Materials and Methods

A cross-sectional analytic study was conducted with MSM. These were members of a sexually diverse community in the Municipality of Manzanillo, Colima, in the western coast of Mexico. Manzanillo is the main international seaport in the Pacific with nearly 200,000 inhabitants, and it is also a major touristic location. Due to these economic activities, it attracts all kinds of visitors, including temporary migrant workers [34].

Manzanillo was chosen as study location because this municipality is home to an organized sexually diverse community of nearly 2,000 members. These individuals are at increased risk of STI and HIV contagion due to their sexual practices in an environment of interaction with migrant groups. In fact, Manzanillo ranked second in terms of HIV-AIDS cases in Colima State in 2018, with incidence rates above the national average [35]. The vulnerability of this community is also manifest in acts of homophobia, and social discrimination and marginalization against its members, which have led authorities to implement public campaigns of information and sensitization in recent years [36].

Study participants were MSM who had lived in the Municipality of Manzanillo within the last three years, aged 18 years or more, and who reported having oral or anal sex with biologic men within the last 12 months regardless of whether they identified themselves as homosexual, bisexual, or transgender woman.

Due to confidentiality concerns, it was not possible to have access to the census of sexually diverse community to have a sampling frame for the selection of a probabilistic sample. Instead, ten MSM who agreed to participate under informed consent were identified with the help of a local non-governmental organization that promotes the prevention and control of HIV/AIDS in this sexually diverse community. Thereafter, those individuals were asked to get in touch with other MSM who they might know, and ask them if they would accept being contacted for possible participation in the study. Those who agreed and provided their contact information were located and requested participation. This snowball sampling technique [37] led to a final sample of 142 MSM who fulfilled the inclusion criteria and agreed to sign the informed consent.

Data were collected using a self-administered questionnaire that included individual background characteristics, psychosocial stressors, experienced stigma, HIV knowledge, knowing someone living with HIV/AIDS, and HIV sexual risk behaviors as follows:

(a) Individual background characteristics included age, educational level, civil status, occupation, type of household, religion, migratory condition, and HIV test history.

(b) Psychosocial stressors included six psychosocial conditions associated with HIV risk in the syndemic (i.e., aggregation of ≥2 concurrent/sequential psychosocial conditions that cause stress in the individual’s life) MSM literature [19], specifically, (1) sexual abuse, (2) imprisonment, (3) intimate partner violence within the last year, (4) sexual work within the previous six months, (5) survival sex practices within the last year, and (6) transgender identity; respondents dichotomously reported the occurrence of such events in their life (yes vs. no).

Social marginalization of the place of residence was the 7th condition added to the list, as this measure of economic inequality reflects the level of social deprivation that has been associated with high rates of late HIV diagnosis [38]. In the Mexican context, this variable is produced by the National Population Council from census data. It is a multidimensional construct that considers the availability of various services such as health, education, household conditions, and access to primary goods. The final index is categorized into urban marginalization levels (i.e., low, medium, high, and very high). For the Municipality of Manzanillo, these levels were dichotomized as 0 = low-medium, and 1 = high-very high [39].

For the analyses, a syndemic index was computed by adding the number of psychosocial conditions (minimum 0, maximum 7). Scores were not computed in participants with missing data for any of the items. If two or more psychosocial conditions occurred simultaneously, a syndemic phenomenon was assumed [26].

(c) Experienced stigma was assessed using a 13-item scale adapted from the Self-Stigma Scale-Short Form [40] and the HIV Stigma Framework [41] covering three stigma mechanisms: cognitive (stereotypes), affective (prejudices), and behavioral (discrimination acts). Each item has a Likert score fluctuating from 1 (never) to 5 (always) for a total scale ranging from 13 to 65 points, with a higher score indicating a stronger sense of experienced stigma. The reliability of this scale in this study was adequate (Cronbach’s alpha = 0.82). For the analyses, scores were categorized as none (13 points), low (14–20 points), and high (≥21 points).

(d) HIV knowledge was assessed using a set of seven items covering transmission routes and modes of prevention commonly used for measuring HIV knowledge in groups at risk [42]. Respondents could answer whether statements were true, false, or unsure. The reliability of this measure was acceptable (Cronbach’s alpha = 0.72). Responses were dichotomized as false/unknown (0 points) vs. true (1 point). The total number of correct answers were added leading to a score between 0 and 7 points. Individuals with 6–7 points were considered with high knowledge, and those with 0–5 point with low knowledge.

(e) Participants were asked if they knew at least one friend/acquaintance living with HIV/AIDS to assess their interpersonal experience with infected individuals using a dichotomous response (yes vs. no). In studies carried out under the theoretical approach of intergroup contact, it has been found that this variable is associated with the development of HIV preventive attitudes and practices [43,44].

(f) To assess sex risk behaviors participants were asked three questions on the following actions regarded as risk factors for HIV transmission among Mexican MSM groups [7]: (1) frequency of condomless anal sex acts with male partners during the past six months rated 1 (never) to 5 (always), defining unprotected sex behavior if rated 1 to 4, (2) frequency of illicit drug use when having sex within the past six months rated 1 (never) to 5 (always), defining risk behavior if rated 2 to 5, and (3) frequency of alcohol use when having sex within the past six months, rated 1 (never) to 5 (always), defining risk behavior if rated 2 to 5.

The self-administered questionnaire was conducted privately, either outdoors or in the participants’ homes once individuals were contacted by community health workers who requested voluntary participation and obtained written informed consent. Data gathered from June to November 2019 was anonymous and confidential. All participants were given preventive information about HIV prevention for MSM. When suitable, participants were referred to a local private assistance institution for counseling, guidance, or clinical care. The study proposal was reviewed and approved by the Research and Ethics Committees at Christus Muguerza del Parque Hospital (ID: HCMP-CEI-20190527-EX01).

Descriptive statistics were used to characterize the study population. Frequencies and percentages were used for nominal data. For variables relating to HIV knowledge and experienced stigma, 95% confidence intervals (CI) of the percentages were estimated using a 1,000-sample bootstrap procedure. The instruments’ internal consistency was calculated using the Cronbach’s alpha coefficient.

For the main analysis, binary logistic regression was used with unprotected anal sex practices during the last six months as dependent variable. Crude and adjusted odds ratios (OR) with 95% CIs, and *p*-values were computed. Independent variables included presence of syndemic stressors (0 = no, 1 = yes), experienced stigma (0 = no stigma, 1 = medium, 2 = high), HIV knowledge (0 = low, 1 = high), interpersonal knowledge of people with HIV (0 = no, 1 = yes), and use of illegal drugs during sex (0 = no, 1 = yes). Variables with a *p*-value <0.10 in crude analyses were selected for adjusted analysis. The model’s goodness-of-fit was assessed using the Hosmer-Lemeshow test; an acceptable fit was indicated by a non-significant Chi^2^ value (*p* > 0.05). All analyses were performed in SPSS v.24.

## 3. Results

Mean ± s.d. age was 30.6 ± 8.5 years (*n* = 142) with 56.3% of individuals aged 18–30 years; 81% were single, and 59.9% had college education or more. All participants reported being religious (Catholic 90.1%, Christian 9.9%) and three out of four were employed. Most lived with their parents/relatives (54.2%) or with a sex partner (29.6%); 15.5% were migrants, and 68.3% self-reported having been tested for HIV (Table 1).

Overall, 52.1% of the respondents gave 6–7 HIV knowledge correct answers out of the seven items asked (*n* = 142). The proportion of correct answers about HIV by item ranged between 57.7% (an infected person can transmit HIV without knowing) and 81% (sharing needles and syringes is a source of HIV infection). Internal consistency for the seven items was relatively high (Cronbach Alpha = 0.72) (Table 2).

Level of experienced stigma was relatively high in MSM (26.1%), yet 33.8% reported no stigma at all. Most frequent discrimination acts reported included work rejection (35.9%), limited access to public places (34.5%), and detention/incarceration (16.9%). Main prejudices included rejection for being regarded as a bad influence (26.8%), and distancing from friends/acquaintances (24.6%). Considering homosexuality contagious (21.8%) and rejection due to possible HIV/STI status (18.3%) were the most common stereotypes reported (Table 3).

Syndemic exposure to psychosocial stressors was present in 49.3% of the sampled MSM. Most frequent stressors were related to situations of violence and poverty. Survival sex within the last year (67.6%), intimate partner violence within the last year (64.5%), sexual work within the last six months (45.1%), and high level of social marginality (38%) were the most common stressors reported (Table 4).

Risky sex practices for HIV infection were common for alcohol use related to sexual intercourse (93%), for unprotected anal sex within the last six months (71.8%), and for drug use related to sexual intercourse (45.8%). 

Table 5 presents crude and adjusted ORs for variables associated with unprotected anal sex practices during the last six months in the surveyed MSM. Five variables showed statistical significance in adjusted analyses (OR; 95% CI). Presence (3–7 events) of syndemic of psychosocial stressors (3.5; 1.25–10), drug use during sex intercourse (3.5; 1.85–6.48), having friends/acquaintances with HIV (2.8; 1.71–4.67), and experienced high (≥21 points) stigma (5.9; 1.42–24.9) were positively associated with unprotected anal sex. Having a high level of HIV knowledge (6–7 answers) was protective against having experienced unprotected anal sex practices (0.4; 0.14–0.96) (Table 5).

## 4. Discussion

The study was conducted to determine if the effect of the syndemic accumulation of psychosocial stressors and experienced stigma were predictors of unprotected anal sex in MSM from the western coast of Mexico. We found that both were independent factors after adjusting for the effect of drug use during sex intercourse, HIV-related knowledge level, and knowing friends or acquaintances with HIV. These findings can be framed within the theoretical approach of stress in sexual minorities associated with the risk of HIV infection [17]. The basic assumption relates to the heteronormative social context that stigmatizes and victimizes the sexual culture of MSM leading to stressful psychosocial experiences with resultant anxiety, depression, substance use, and HIV risk practices.

The associations found match the findings of empirical studies that reported both, the dose-response effects of exposure to syndemic of stressors, and the exposure effects to high levels of experienced stigma in the context of a higher risk of unprotected anal sex practices in MSM where statistical significance remained after adjustment of other risk (e.g., substance use) and protective (e.g., HIV knowledge) factors [20,21,22,23,24,25,26].

Knowing people with HIV/AIDS was another relevant risk factor identified. This result relates to the influence of intergroup contact on cognitive processes associated with the perception of risk and identity of infected people [44]. Different studies have shown that knowing people with HIV in a stigmatizing social context can lead to the development of empathetic attitudes with less prejudice towards those infected, and to the adoption of practices as the infected, pointing to the predominant adoption of unprotected anal sex practices [43,45].

The syndemic presence of psychosocial stressors also show that nearly half of the MSM interviewed were exposed to high levels of stressful events, and that two out of three experienced stigma potentially increasing the risk of HIV transmission [19,29,30,31] in consonance with the high proportion of reported unprotected anal sex and use of illegal drugs during sexual intercourse (45.8%).

In terms of study limitations, five main issues must be stressed. First, the cross-sectional nature of the design precludes strong causal explanations; however, longitudinal evidence has also shown the impact of psychosocial syndemic variables as predictors of sexual risk practices in MSM [46]. Second, the snowball sampling strategy used restricts the probabilistic representativeness of the results; yet this approach had to be used, as there was no community census of MSM available. Third, the syndemic analysis of stressors was limited to the dose-response cumulative approach; future studies could also use and combine the synergistic interaction to assess the syndemic effects comparatively and complementarily. For instance, some studies have focused on the effect of the synergistic interaction of syndemic stressors on the risk of unprotected anal sex practices among MSM showing significant associations when the interactions between stressors included alcohol and illicit drug use, partner violence, and depressive conditions [47], as well as the perception of discrimination and illegal drug consumption [48], and the history of childhood sexual abuse, and frequent alcohol use [49]. Fourth, the analysis of stigma could be amplified to better understand its structural and interpersonal effects. North American studies show that various forms of stigma against MSM contribute to generate barriers to engage in preventive activities, decrease the adherence to treatments, and lower the access to health services in MSM regardless of HIV status. When MSM feel discriminated against by health care personnel, awareness of the importance of using protection in their sexual practices decreases [50]. Evidence from European countries and in cities in the United States with high levels of structural stigma also found indicators of vulnerability to HIV in MSM, such as high prevalence of risky sexual practices, and less access to HIV diagnostic tests [51,52]. Finally, the dependent variable used was limited to unprotected anal sex during the last six months; while this practice is considered the main risk factor for HIV in MSM, a composite variable that considers various risk practices (e.g., a standardized risk index) could lead to a more accurate assessment.

## 5. Conclusions

This study shows that high exposure to psychosocial stressors along with high levels of experienced stigma are independent risk factors of unprotected anal sex in MSM in that coastal region of Mexico, contributing to the vulnerability of sexual transmission of HIV. The findings highlight the relevance of the meaningful integration of mental health services in conjunction with stigma-mitigation strategies as part of a broader strategy to reduce sexual HIV transmission among Mexican MSM.

## Figures and Tables

**Table 1:** Sociodemographic characteristics of the interviewed men who have sex with men, Manzanillo, Mexico.

Variable	Category	Frequency	Percent
Age	18–30	80	56.3
	31–40	41	28.9
	41–50	21	14.8
Schooling	Primary and high school	57	40.1
	College degree and more	85	59.9
Civil status	Single	115	81.0
	Cohabiting	27	19.0
Main occupation	Student	21	14.8
	Employee	105	73.9
	Both, student/employee	9	6.4
	Unemployed	7	4.8
Religion	Catholic	128	90.1
	Christian non-Catholic	14	9.9
Type of household	Lives with parents/relatives	77	54.2
	Lives with a sex partner	42	29.6
	Lives alone or with friends	23	16.2
Migratory situation in the municipality	Native to the place	120	84.5
	Migrant	22	15.5
Friends/acquaintances living with HIV	Yes	83	58.5
	No	59	41.5
Self-report of having been tested for HIV	Yes	97	68.3
	No	45	31.7
Total		142	100.0

**Table 2:** HIV-related knowledge ^1^ in interviewed men who have sex with men, Manzanillo, Mexico (*n* = 142).

HIV Questions Asked	Correct Answers ^2^		Cronbach’s α
	Percent (*n*)	95% CI ^3^	
An infected person can transmit HIV without knowing it	57.7 (82)	49.3–66.2	0.72
Sharing needles and syringes can infect HIV	81.0 (115)	74.6–86.6	
Using a condom can protect against the risk of contagion	73.2 (104)	65.5–80.3	
Reducing the number of partners prevents HIV risk	58.5 (83)	50.7–66.9	
Sharing dishes with a sick person can infect HIV	70.4 (100)	62.0–77.5	
Anal sex carries a high risk of HIV infection	69.0 (98)	60.6–76.8	
Patients with STIs are more vulnerable to HIV infection	76.8 (109)	69.0–83.1	

^1^ Low: 1 to 5 correct answers = 47.9%; high: 6 to 7 correct answers = 52.1%. ^2^ According to conventional epidemiological criteria. ^3^ Bootstrap for percentage of 1000 samples.

**Table 3:** Experienced stigma ^1^ among interviewed men who have sex with men, Manzanillo, Mexico (*n* = 142).

Dimensions and Items	Presence of Stigma ^2^		Croncbah’s α
	Percent (*n*)	95% CI ^3^	
Discrimination acts			0.82
Denied work/employment access	35.9 (51)	27.5–43.7	
Denied access to public places	34.5 (49)	26.8–43.0	
Detained or jailed	16.9 (24)	11.3–23.2	
Denied registration in a school	13.3 (19)	7.7–19.0	
Denied participation in social organizations	8.5 (12)	4.2–13.4	
Denied access to health services	4.9 (7)	1.4–8.5	
Prejudices			
Rejected because you were considered a bad influence	26.8 (38)	19.0–33.8	
Friends/acquaintances distanced themselves from you	24.6 (35)	17.6–31.7	
Limited participation to religious events	15.5 (22)	9.9–21.8	
Expelled from home or residence place	15.5 (22)	9.9–21.8	
Stereotypes			
Rejected because homosexuality can be contagious	21.8 (31)	15.5–28.2	
Rejected because you might have HIV or a STI	18.3 (26)	12.0–23.9	
Rejected because you don’t deserve equal treatment	17.6 (25)	11.3–23.9	

^1^ No stigma (score ≤13) = 33.8%, low stigma (score 14–20) = 40.1%, high stigma (score > 20) = 26.1%; ^2^ Includes scores from 2 to 5 in a Likert scale (at least once, sometimes, nearly always, always); ^3^ Bootstrap for percentage of 1000 samples.

**Table 4:** Exposure to psychosocial stressors ^1^ of the interviewed men who have sex with men, Manzanillo, Mexico.

Variable	Category	Frequency	Percent
Transgender identity	Yes	34	23.9
	No	108	76.1
Level of social marginality ^2^	High	54	38.0
	Medium/low	88	62.0
Sexual work experience within the last six months	Yes	64	45.1
	No	78	54.9
Survival sex experience within the last year	Yes	96	67.6
	No	46	32.4
Intimate partner violence within the last year	Yes	66	64.5
	No	76	53.5
Sexual abuse experience	Yes	32	22.5
	No	110	77.5
Imprisonment experience	Yes	24	16.9
	No	118	83.1

^1^ Syndemic exposure to psychosocial stressors: yes (3–7 events) = 49.3%; no (0–2 events) = 50.7%; ^2^ This variable was added to the syndemic scale when individuals had high marginality.

**Table 5:** Crude and adjusted odds ratios from binary logistic regression for variables associated with unprotected anal sex practices during the last six months in sureveyed men who have sex with men in Manzanillo, Mexico, 2019.

Variables	Unprotected anal Sex *n* (%)		Odds Ratios (95% Confidence Intervals)	
	Yes (*n* = 102)	No (*n* = 40)	Univariate	Multivariate ^1^
Syndemic of psychosocial stressors				
No (0–2 events)	45 (62.5)	27 (37.5)	1.00	1.00
Yes (3–7 events)	57 (81.4)	13 (18.6)	2.6 (1.22–5.67) *	3.5 (1.25–10.0) *
Experienced stigma (score)				
No stigma (≤13)	26 (70.3)	11 (29.7)	1.00	1.00
Low (14–20)	35 (72.9)	13 (27.1)	1.0 (0.43–2.69)	1.2 (0.34–4.34)
High (≥21)	41 (71.9)	16 (28.1)	1.1 (0.44–2.94)	5.9 (1.42–24.9) *
Drug use related to sex intercourse				
No	47 (61.0)	30 (39.0)	1.00	1.00
Yes	55 (84.6)	10 (15.4)	3.5 (1.55–7.92) **	3.5 (1.85–6.48) ***
HIV-related knowledge level				
Low (1–5 correct answers)	53 (77.9)	15 (22.1)	1.00	1.00
High (6–7 correct answers)	49 (66.2)	25 (33.8)	0.5 (0.26–1.17)	0.4 (0.14–0.96) *
Friends/acquaintances with HIV				
No	32 (54.2)	27(45.8)	1.00	1.00
Yes	70 (84.3)	13(15.7)	2.1 (1.43–3.11) ***	2.8 (1.71–4.67) ***

* *p* < 0.05, ** *p* < 0.005, *** *p* < 0.001; ^1^ Hosmer & Lemeshow adjustment dependability test *p*-value = 0.25.

## Data Availability

Not applicable.
